# Crossed patterned structured illumination for the analysis and velocimetry of transient turbid media

**DOI:** 10.1038/s41598-018-30233-y

**Published:** 2018-08-06

**Authors:** Elias Kristensson, Edouard Berrocal

**Affiliations:** 1 Department of Physics, Division of Combustion Physics, Lund University, Lund, Sweden; 2 Erlangen Graduate School in Advanced Optical Technologies (SAOT), Universität Erlangen-Nürnberg, Erlangen, Germany

## Abstract

Imaging through turbid environments is experimentally challenging due to multiple light scattering. Structured laser illumination has proven to be effective to minimize errors arising from this phenomenon, allowing the interior of optically dense media to be observed. However, in order to preserve the image spatial resolution while suppressing the intensity contribution from multiple light scattering, the method relies on multiple acquisitions and thus sequential illumination. These requirements significantly limit the usefulness of structured illumination when imaging highly transient events. Here we present a method for achieving snapshot visualizations using structured illumination, where the spatial frequency domain is increased by a factor of two compared to past structured illumination snapshots. Our approach uses two crossed intensity-modulated patterns, allowing us to expand the spatial frequency response of the extracted data. The snapshot capability of this imaging approach allows tracking single particles and opens up for the extraction of velocity vectors by combining it with standard particle tracking/image velocimetry (PTV or PIV) equipment. In this paper we demonstrate the capabilities of this new method and, for the first time, use structured illumination to extract velocity vectors in 2D in a transient turbid medium, in this case an optically dense atomizing spray.

## Introduction

Atomizing sprays form the base for many combustion engine concepts commonly used in modern society, such as gasoline direct injection (GDI) engines, gas turbines and Diesel engines. Analyzing and characterizing such spray systems is very important to both optimize the combustion efficiency and reduce the emission of pollutants. However, the highly transient and turbid nature of those systems makes this task extremely challenging^1–6^. An ideal probing technique for spray studies should be non-intrusive and provide two- or three-dimensional spatially resolved data, with a temporal resolution of a few hundreds of nanoseconds. In addition, the technique should provide velocity vectors as well as being able to selectively probe either the liquid- or the gas phase. Currently, light-based diagnostic techniques are the only candidates with the potential of fulfilling all these requirements. However, most optical imaging methods suffer from measurement errors caused by multiple light scattering occurring inside the dense cloud of droplets/ligaments that constitutes the spray. When light propagates through a cloud of scattering particles, a large portion of the photons interact multiple times with the sample before exiting^2^. Since most light-based imaging techniques rely on the single scattering approximation, detecting such multiply scattered light leads to qualitative as well as quantitative measurement errors^3,4,7^. Qualitatively, the detection of multiply scattered light leads to image blur and a degraded image contrast^1,2,8,9^. Quantitatively, it leads to erroneous intensity levels as well as deviations from theoretical predictions^10^. Within the last three decades, much effort has been made on developing new diagnostic tools in order to solve these issues. Means to temporally filter the light being transmitted through a scattering environment – a concept known as Ballistic Imaging (BI) – have been developed and employed by several research groups^1,7,9,11–15^. Spatial Fourier filter can also improve visibility in scattering environments as shown by Alfano *et al*.^16^. In addition to the use of visible light, X-rays are also used to study atomizing sprays, where the weak interaction between X-rays and matter is exploited^17^. By adding a fuel tracer, the integrated X-ray absorption through a spray can be quantified, which holds information on the liquid mass^18^. Recently, high-speed X-ray imaging of an atomizing spray has also been demonstrated^19^. Unfortunately, strategies relying on temporal gates, spatial filtering and X-rays are restricted to line-of-sight optical configurations and are therefore not unifiable with optical sectioning configurations such as laser sheet-based imaging techniques (side scattering methods).

## Structured Illumination (SI)

Another experimental approach to solve issues related to multiple light scattering in optically dense environments is to use a structured light source – a technique known as Structured Illumination (SI)^20–22^. One benefit of this particular approach over the previously mentioned ones is its versatility; applicable for transmission detection^22^, back- and side scattering^20,22–24^, 3D imaging^25^ as well as for elastic- and inelastic scattering^10^. Another important aspect of SI is that is does not rely on expensive optical hardware or the use of an X-ray synchrotron. Structured Illumination uses a light source that has a sinusoidal pattern imprinted in its intensity profile. The purpose of the pattern is – when applied for imaging turbid media – to distinguish between the desired signal and the light that was multiply scattered inside of the probed medium. This spatial code is maintained by the unperturbed photons (ballistic light) when detected in the forward direction (transmission configuration) and by the singly scattered light when detected on the side (light sheet imaging) or in the backward direction.

Structured Illumination has, however, one limitation that renders snapshot realizations challenging: it relies on multiple acquisitions and thus sequential illumination^20,22,24^. This requirement is due to the fact that not all parts of the sample are irradiated in a single illumination event and sample information from the regions “between the lines” needs to be acquired sequentially. Imaging rapidly evolving on-time events, such as high pressure atomizing sprays, is thus technically challenging with SI, demanding advanced hardware that can deliver and acquire a sequence of laser pulses within ~100 ns^26^. Extracting velocity information using e.g. Particle Image Velocimetry (PIV)^27–29^, which in turn is also based on sequential illumination, thus becomes particularly challenging. As a result, velocity mapping of rapidly evolving samples based on SI has, to the best of the authors’ knowledge, not been achieved yet.

Recently, means to estimate the contribution from unperturbed/singly scattered light from a single modulated “subimage” (one-phase Structured Illumination, or 1p-SI) has been presented for snapshot imaging^30,31^, holding promise to solve this particular issue (note that “phase” in one-phase Structured Illumination refers here to the use of a single sinusoidal intensity modulation and not to the different states of matter). The analysis of such a 1p-SI method is based on a spatial frequency-sensitive algorithm, where a band-pass filter is applied (in the Fourier domain) on the fundamental frequency component of the superimposed intensity modulation. The main drawback, however, with currently presented 1p-SI methods concerns the loss in spatial resolution, since it only extracts a small portion of the spatial frequencies offered by the imaging system.

In this paper we present a snapshot SI imaging approach, referred to as xSI (crossed Structured Illumination), that maintains both the filtering capabilities offered by 1p-SI and good spatial resolution. The approach is further implemented on a light sheet imaging configuration where it is referred as xSLIPI (crossed Structured Laser Illumination Planar Imaging). We demonstrate, here, the applicability of xSLIPI for obtaining both high contrast and good spatial resolution snapshot images of a turbid atomizing spray. Finally, we exploit the snapshot feature to record double-exposure images to extract the velocity field of the droplet cloud from an optically dense atomizing spray system.

## Theory

**Figure 1:**
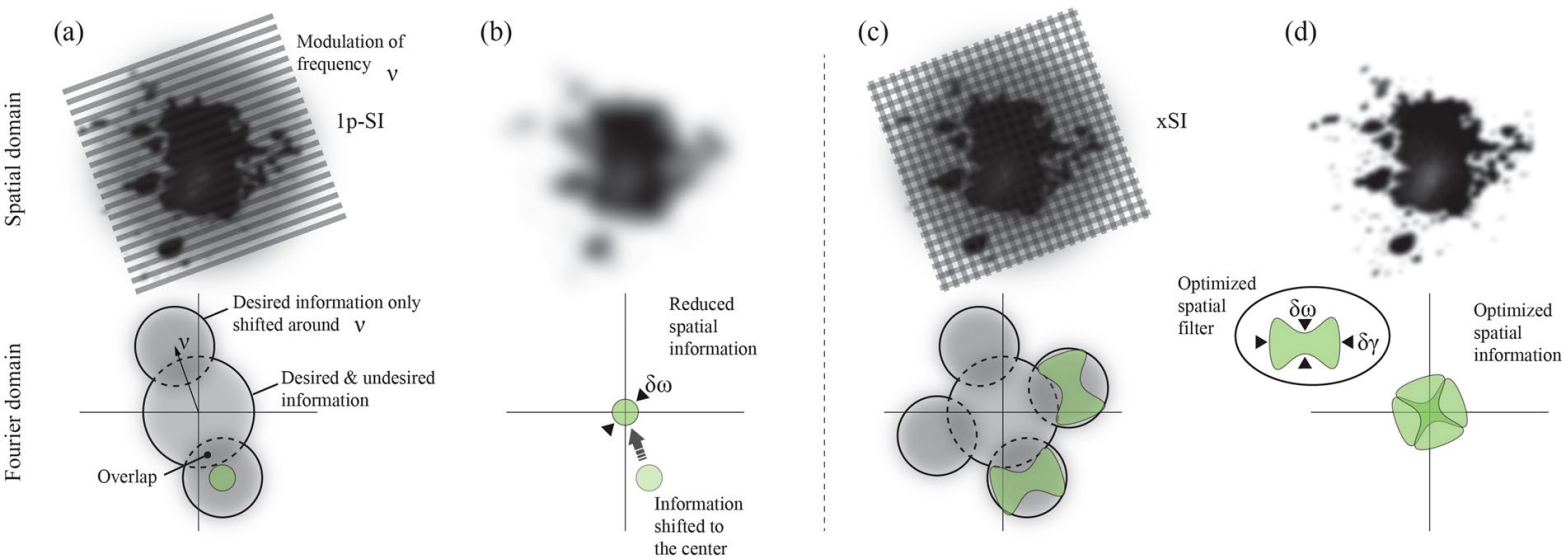
The principle of xSI. (**a**) A turbid sample illuminated with a single intensity-modulated source. (**b**) Lock-in analysis of the image in (**a**) removes the background blur but renders poor image resolution due to the limited bandwidth of the band-pass filter (δω). (**c**) Two crossed intensity-modulated sources simultaneously illuminating a turbid sample. The signal from each source provides relatively high spatial frequencies in different directions in reciprocal space. (**d**) Spatial frequencies in (**c**) merged into a single image, having improved spatial resolution compared to (**b**).

To improve the spatial resolution of an imaging system, the frequency response, in the Fourier domain, needs to be expanded. To avoid misinterpretations regarding sample structures, this response should be uniform, i.e. identical spatial resolution in both lateral directions. Figure 1 illustrates the principle of xSI and how it differs from the past snapshot SI method (1p-SI), where a single line pattern is used. A sample illuminated with a single sinusoidal intensity-modulated light field of frequency ±ν creates two “image copies” of the sample structures at ±ν (Fig. 1a)^32^. Thus the information residing within these regions comes solely from the illumination, i.e. the desired information. At lower frequency, however, the central region contains not only the desired signal but also unwanted intensity contributions, such as surrounding light, indirect reflections, multiply scattered light, backgrounds, *etc*. By applying a frequency-sensitive spatial lock-in algorithm, the information within the fundamental frequencies (±ν) can be accessed (Fig. 1b) while the low frequency data is suppressed. However, the dimensions of the band-pass filter (marked in green) used in the lock-in algorithm are restricted by (1) the desire to achieve a uniform frequency response and (2) the spread of the central (low frequency) region, resulting in an image with good specificity but with relatively poor spatial resolution. This methodology – 1p-SI – thus leaves large portions of the reciprocal space unexploited.

To better exploit the available reciprocal space, xSI employs a multiplexed illumination strategy, where two crossed intensity-modulated sources simultaneously irradiate the sample (Fig. 1c). Compared to the 1p-SI approach, this produces two additional image copies of the sample structures that are created in two other regions of the Fourier domain. Note, however, that both sets of image copies are identical and contain the same information. This allows us to apply a spatial lock-in algorithm on each set of image copies individually with non-uniform band-pass filters, each specifically designed to extract complementary spatial frequencies that could not be accessed with a single modulation due to signal cross-talk. The procedure yields two snapshot images that then are merged into one with an expanded, yet still uniform, frequency response in comparison with the 1p-SI method (Fig. 1d). In other words, since the intensity-modulated sources are orthogonal, the information that is lost from the regions “between the lines” is thus minimized, which essentially yields the improvements in spatial resolution of xSI over 1p-SI.

Experimentally, xSI does not require additional- or more advanced hardware (lasers/detectors) than 1p-SI. Both modulated light fields are recorded within a single camera exposure. The crossed illumination pattern can be accomplished from a single laser source, by simply splitting the laser beam into two channels using beam-splitter.

## Experimental Setup

Two experimental arrangements were used in this study. Initially, a validation experiment was performed using a transmission-based configuration to investigate the improvement in spatial resolution xSI offers, while for the spray- and velocity measurements a planar configuration named xSLIPI was constructed and tested for a challenging measurement situation involving high velocities and turbidity.

### Validation experiment

**Figure 2:**
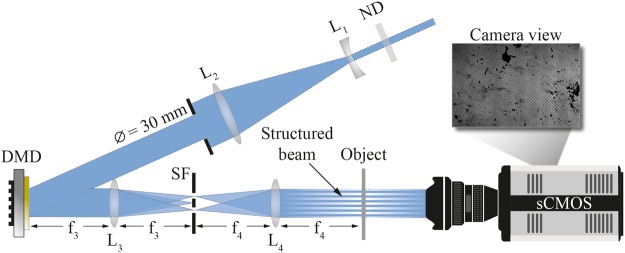
Experimental setup used to validate the xSI approach. A DMD (V-9501 VIS) was used to project two crossed intensity-modulated sine patterns onto the sample of interest. L_1_ = −100 mm, L_2_ = 300 mm, L_3_ = 150 mm, L_4_ = 300 mm, SF = spatial filter, ND = neutral density filter with an optical density of 2.0.

To investigate the performance of xSI a transmission-based SI optical setup was constructed, see Fig. 2. In the setup a Diode Pumped Solid State (DPSS) continuous-wave laser (λ = 447 nm) illuminated a Digital Micromirror Device (DMD), creating the cross-modulated patterns. The DMD was a Vialux V-9501 VIS model consisting of 1920 × 1080 micro-mirrors with a switching rate of 10 kHz. The modulated patterns, projected by the DMD, were then imaged onto a sample using a 4 f optical configuration and the transmitted light was imaged by a 5.5 mega-pixel sCMOS camera (Zyla, Andor Technology). Note that this validation study focused only on investigating whether spatial resolution could be improved by means of xSI and not its ability to suppress multiply scattered light. For that reason, the sample under study here did not contain any turbid elements. Instead it consisted of soot aggregates deposited onto a glass plate, where the aggregates contained structural details that a “traditional” 1p-SI system would fail to resolve.

**Figure 3:**
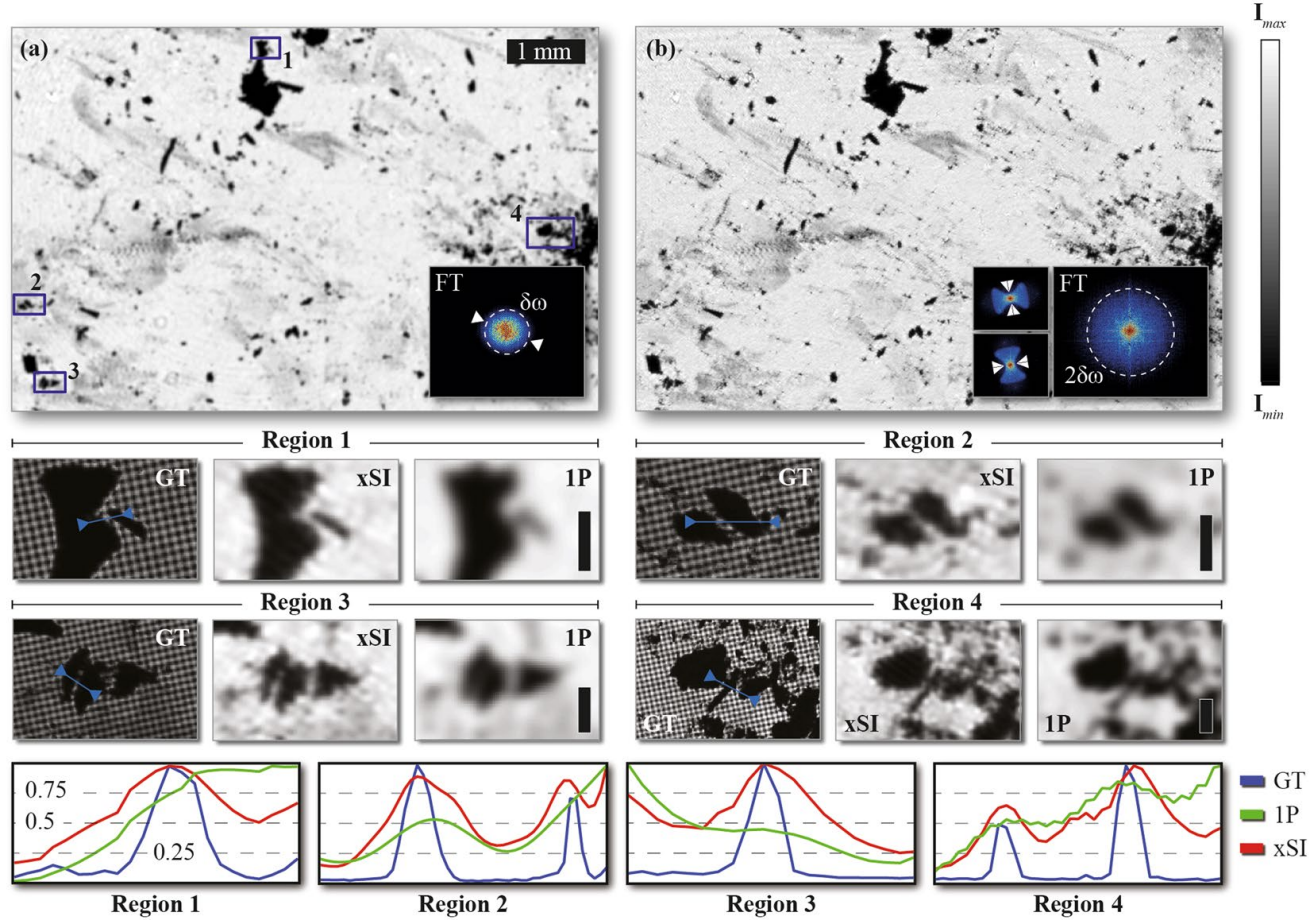
Experimental validation of the improved spatial resolution achievable with xSI. 1p-SI imaging (**a**) and xSI (**b**) of soot deposits (light-blocking objects) together with their corresponding 2D Fourier transforms (FT). Note the increase in observable spatial frequencies in the Fourier domain in the latter case. Also included are four magnified regions, wherein the improved resolution of xSI over 1p-SI can be observed (scale-bars equal to 100 µm). Ground-truth (GT) comparisons are also provided here, comprising of unprocessed data. The spatial resolution in these GT images is thus the highest achievable with the current imaging system. The line-plots extracted from the different regions further display the ability of xSI in observing finer details, all of which are supported by the GT plots.

To enable a fair comparison between 1p-SI and xSI, the analysis of the two images had two constraints. First, the final image should have a uniform response of spatial frequencies. Second, the δω parameter of the band-pass filters in the lock-in algorithm should be equal (see Figs 1 and 3), i.e. the parameter dictating the available bandwidth of spatial frequencies along the ν vector. Exceeding this bandwidth leads to cross-talk between the central cluster of spatial frequencies. For e.g. spray visualization, this would lead to the inclusion of multiply scattered light in the lock-in analysis, often observed as residual lines in the final 1p-SI image^33^.

**Figure 4:**
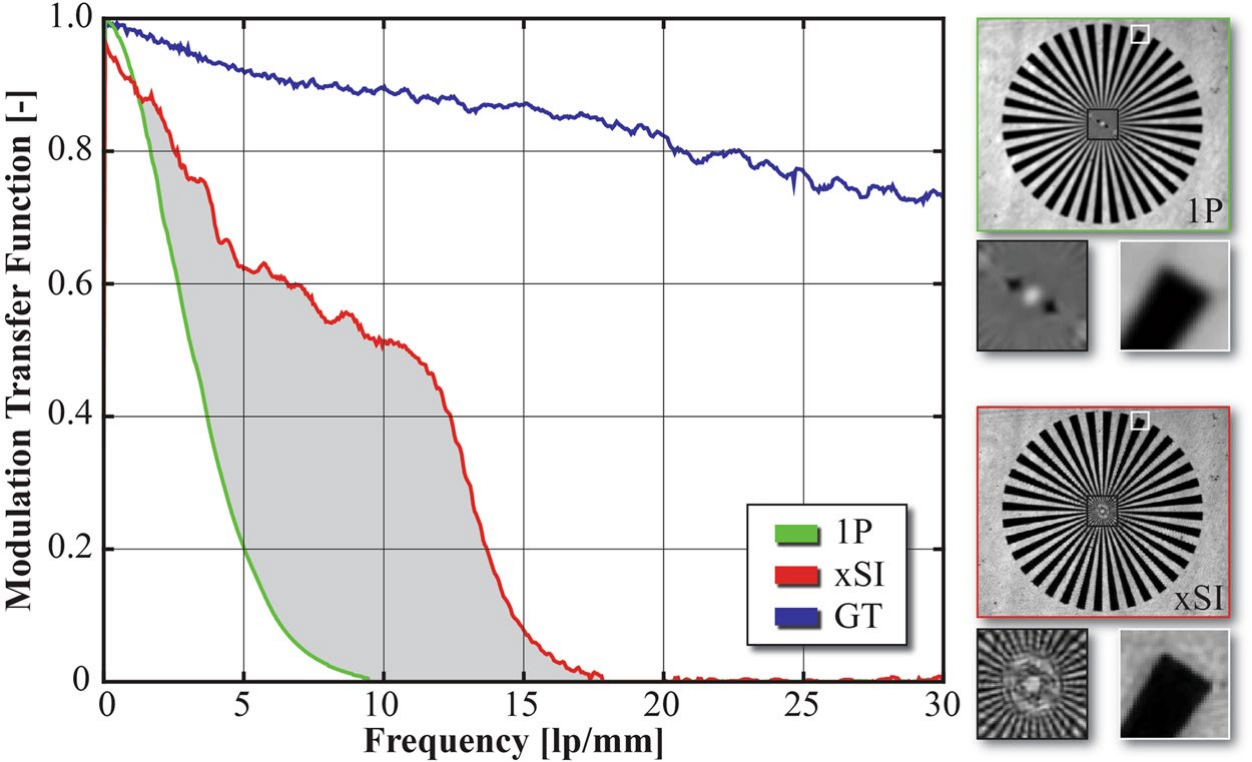
Comparison between 1P-SI, xSI and ground truth (GT) through experimentally measured Modulation Transfer Functions (MTFs), together with images of the resolution target used to determine the MTFs.

Figure 3 shows one example of the results from the validation measurements. In Fig. 3a the outcome of the 1p-SI analysis is given while the corresponding xSI image is displayed in Fig. 3b. The insets in each panel show the corresponding 2D Fourier transform, in which the cut-off frequency is marked. Notice that the cut-off frequency in the xSI image is approximately twice as large. Also included in the figure are four magnified regions-of-interest, wherein the improved spatial resolution achieved with xSI is more apparent. The improved spatial resolution is further illustrated in the line-plots in Fig. 3, supported by line-plots extracted from ground-truth (GT) data. Figure 4, which presents experimentally measured Modulated Transfer Functions (MTFs), also illustrates the improved spatial resolution with xSI.

The most computationally time demanding operation when extracting a 1p-SI image is the two-step Fourier transformation which is required^34^. Since xSI relies on extracting at least one additional image information from the Fourier domain and one additional Fourier transformation, it becomes slightly more computationally demanding than 1p-SI. Furthermore, in order to merge the two images (in the xSI case) into one with improved resolution, the images need to be field-dependent normalized, which is a standard approach for structured illumination, see ref.^35^. This procedure compensates for low frequency variations between the images, which could arise due to instabilities in the illumination (especially for SI systems based on sequential illumination). This normalization procedure also requires one Fourier- and one inverse Fourier transformation, which adds to the overall computational time. For the measurements presented in the current proof-of-concept study, which were based on recording images of 2560 × 2160 pixels, the typical time for extracting a 1p-SI image using MATLAB was about one second, while the corresponding time for an xSI image was almost four seconds. The computational time could, however, be reduced by approximately a factor of two by determining the phase of the intensity modulation which is used as input into the spatial lock-in algorithm to access the modulated information in the Fourier domain (see Appendix A in ref.^36^.). However, note that measuring the phase is not always straightforward and depends on the measurement situation.

### Setup for spray visualization

In the case of sprays, as well as most other flow systems, light sheet imaging is usually more beneficial than line-of-sight imaging approaches as it provides sectioned views of the three-dimensional flow structure. Here, a light sheet imaging configuration based on xSI is tested on an optically dense atomizing spray. As the concept of using structured illumination in planar imaging is a technique known as SLIPI^8^, the approach used in this section is referred to as xSLIPI.

**Figure 5:**
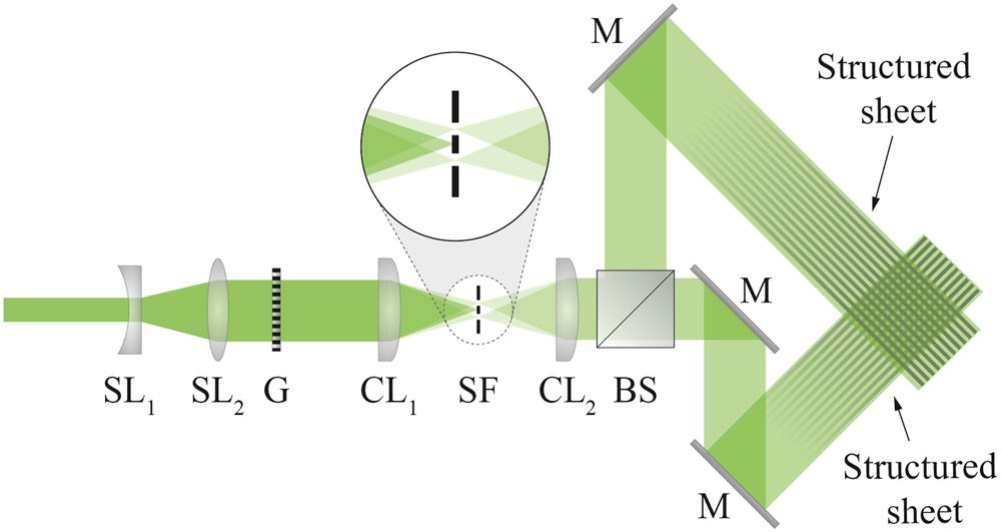
Experimental setup. Collimated light is guided through a Ronchi grating (G) with 30 lp/mm and thereafter focused with a cylindrical lens (CL_1_) onto a spatial filter (SF), permitting only the ±1 orders of diffraction to pass. A cylindrical lens (CL_2_) forms the light into a sheet in the plane of the drawing. A 50/50 beam-splitter (BS) splits the light into two pulses, each redirected to illuminate the spray from different angles. SL_1_ = −100 mm, SL_2_ = 300 mm, CL_1_ = 300 mm, CL_2_ = 1000 mm, M = mirror.

Although the basic principle for xSLIPI is the same as for the transmission-based experiment, a planar configuration does require some additional alignment considerations. Figure 5 shows the optical setup in our proof-of-concept study. In the setup, a Ronchi grating (30 lp/mm) was illuminated by a pulsed Nd:YAG laser (λ = 532 nm). A cylindrical lens relayed the pattern onto the spray. A spatial filter was employed to only permit the ±1 orders of diffraction – a common approach in SI to produce a pure sinusoidal intensity modulation^21,37^. Thereafter a second cylindrical lens focused the light into a thin sheet of light. To enable xSLIPI, the light was guided through a beam-splitter and directed onto the sample from different angles using a set of three mirrors. Note that the beam-splitter and the mirrors are the only optical components necessary to modify a SLIPI setup to xSLIPI. Proper alignment of the two laser sheets is crucial as these need to propagate in the same plane. In addition, the optical path length from the sheet-forming lens should be approximately the same.

## Results and Discussion

### Spray visualization

**Figure 6:**
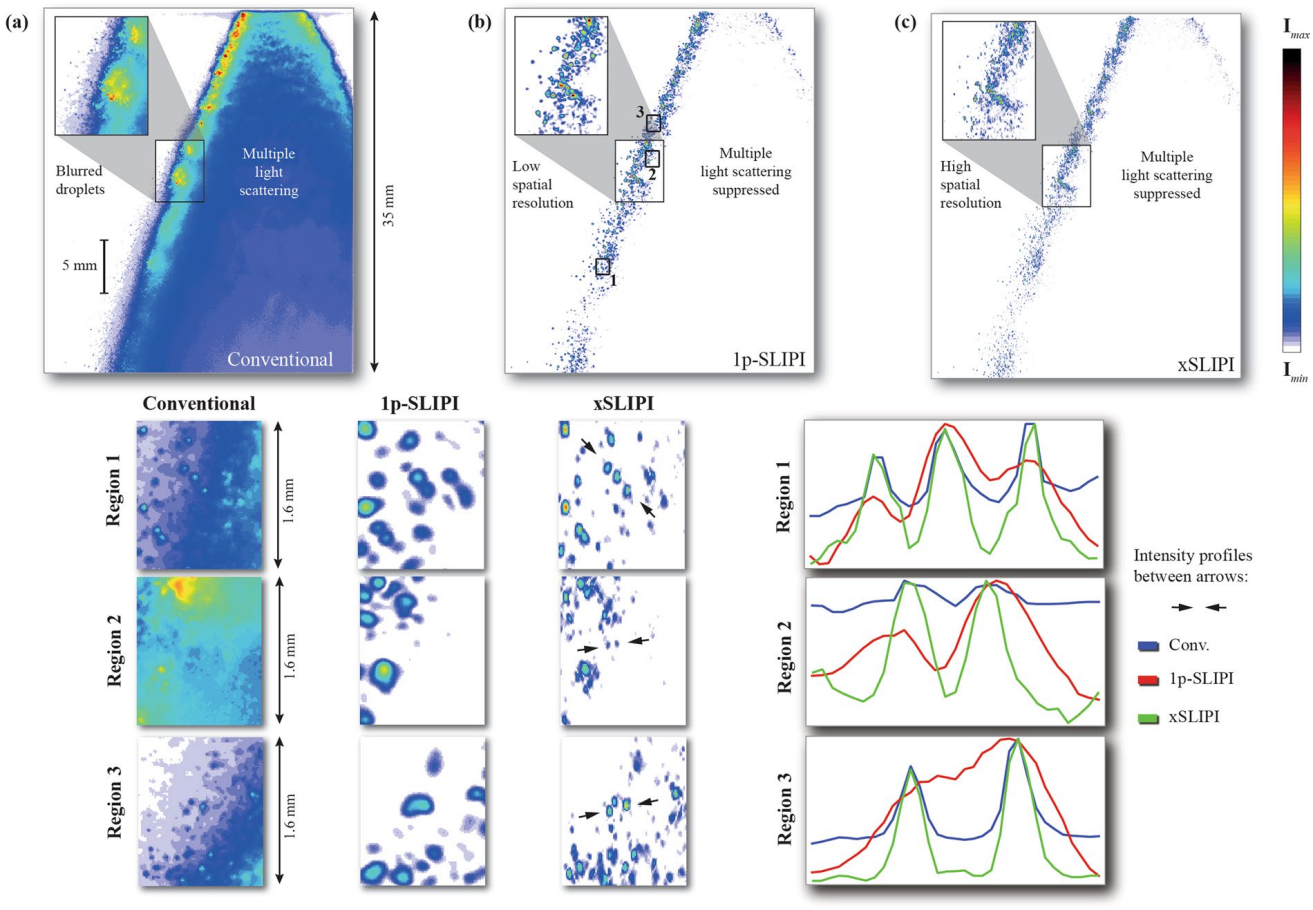
Single-shot measurements of a water spray at 150 bars. (**a**) Conventional laser sheet imaging, (**b**) 1P-SLIPI imaging and (**c**) xSLIPI. The subpanels below each respective image show three magnified regions, where the arrows indicate the end points of the line-plots provided to the right of the figure. Based on these line-plots, Michelson contrast values are calculated (see Table 1).

The spray under study was a hollow-cone steady-state water spray doped with dye EosinY, which produces a fluorescence signal with a quantum yield of 0.67 when excited at 532 nm. Note that liquid fluorescence is of interest here as it provides a much more faithful representation of the liquid structures than the elastic scattering signal does^38^. The liquid was injected up to 150 bars injection pressure allowing a very efficient liquid atomization. To detect the fluorescence signal a 5.5 megapixel sCMOS dual-frame camera (Imager sCMOS, Lavision GmbH), equipped with a notch filter to reject the elastically scattered light, was employed. The dual-frame imaging capability of the camera allowed for a minimum time separation between the two frames of around 200 ns. To detect any displacements of the droplets between the two illumination events, a value of 1.5 μs was used in this experiment. Figure 6 shows single-shot measurements of the spray injected with a pressure of 150 bars for conventional laser sheet imaging (camera view), 1p-SI and xSI. Note that since both modulated light fields illuminate the sample simultaneously, the illumination time is not increased and equals ~8 ns. The experimental challenge of visualizing such an optically dense cloud of droplets is apparent in the conventional acquisition, where the multiply scattered light illuminates off-axis planes. One of the main concerns with this phenomenon is that it reduces the optical sectioning capability significantly. A direct consequence of this reduction is that the spray does not appear hollow, which is in agreement with previous observations of hollow-cone sprays^8,26^. The insets also highlight how the poor optical sectioning strength makes the liquid sheet appear thicker due to the inclusion of scattered light from liquid structures in adjacent planes. Such contributions may lead to e.g. false interpretations related to the atomization process. For example, if the observed signal is assumed to originate from within the ~100 μm thick laser sheet plane, the number density of liquid bodies/droplets would be largely overestimated. By filtering out the out-of-plane signal contribution, 1p- and xSLIPI both reveals a more diluted spatial distribution of liquid bodies and droplets. However, this filtering process comes at the cost of spatial resolution as can be seen in the 1p-SLIPI case in Fig. 6(b), where isolated droplets either cannot be resolved or falsely appear larger in size. The aim of the xSLIPI technique is to reduce such losses in image resolution to achieve a spatial resolution near that of the conventional image (Fig. 6(c)) while still suppressing the large intensity contribution from multiple light scattering. Resolving isolated droplets requires a larger bandwidth of spatial frequencies compared to the past one-phase based structured illumination method.

To determine the performance of xSLIPI in terms of spatial resolution and image contrast, three regions containing closely situated fine droplets were selected (see Fig. 6).

- Region 1 contains three fine droplets, resolvable by all three methods.
- Region 2 contains two droplets that 1P-SLIPI fails to resolve.
- Region 3 contains two droplets that are barely resolvable with conventional imaging.

Table 1 shows estimated values of the Michelson contrast as well as the measured Full Width at Half Maximum (FWHM) of the droplets for all three methods and in all regions, evaluated using the line-plots in Fig. 6. The Michelson contrast was calculated based on the two most intense peaks. The analysis shows that xSLIPI provides the highest contrast for all three cases. In region 3, which contains a significant amount of contribution from multiply scattered light, the contrast is boosted from 9% with conventional imaging to 62% with xSLIPI. In region 2, 1P-SLIPI fails to resolve the two droplets, while the expanded spatial frequency response provided by the xSLIPI analysis permits them to be resolved, with a Michelson contrast of 87%. In the field of spray visualization, SI has thus far focused mostly on removing undesired background contributions that lead to erroneous intensity levels. However, the current analysis of the FWHM in Table 1 shows that xSLIPI provides results with similar spatial resolution as conventional imaging, when applied to turbid, scattering media.

**Table 1:** Michelson contrast values for line-plots in Fig. 6. The values are calculated based on the minimum value in-between two peaks.

		Region 1	Region 2	Region 3
Contrast	Conventional	36%	52%	9%
	1P-SLIPI	24%	—	42%
	xSLIPI	79%	87%	62%
Width	Conventional 1P-	2.6 px	3.0 px	4.9 px
	SLIPI	4.3 px	6.8 px	—
	xSLIPI	2.6 px	3.3 px	3.4 px

### Velocity fields

Due to the snapshot capacity of the xSI technique it can easily be combined with double-pulsed planar PIV methods in order to assess the velocity field of the droplets. To enable this in our current experiment, we aligned two pulsed Nd:YAG lasers to spatially overlap their beams. Combining the beams was achieved using a frequency-doubling scheme, where the first frequency-doubled beam is combined with the fundamental beam of the second one. The two beams were then routed through a Second Harmonic Generating (SHG) crystal in order to convert the second beam (1064 nm) into 532 nm. For detection of the two planar Laser-Induced Fluorescence (PLIF) images., the sCMOS camera ran in double-frame mode with a ∆t = 1.5 μs. With this value of ∆t a droplet traveling with a downwards velocity of 100 m/s becomes vertically displaced ~5 pixels, which was deemed a good match with the expected range of velocities (up to 200 m/s). However, this choice affects the ability of quantifying the velocity of slow-moving droplets, where velocities below ~20 m/s will most likely not be accurately registered.

**Figure 7:**
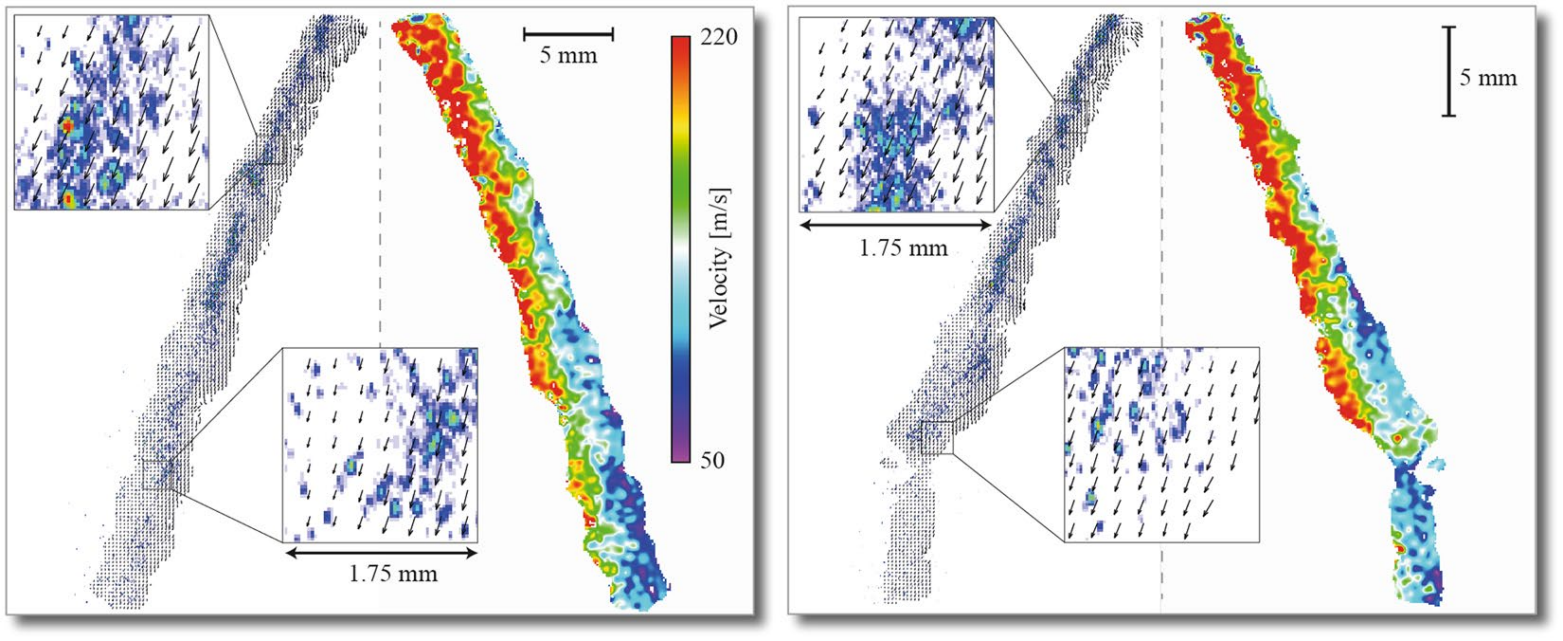
Velocity fields extracted using the PIV-xSLIPI setup, liquid pressure of 150 bars. Velocity vectors are presented to the left in each image whereas the velocity map is presented to the right.

Figure 7 shows two examples of typical PIV results from the hollow-cone water spray obtained using the double-pulsed xSLIPI setup. The left-hand panel in each graph shows the velocity vectors while the velocity map is shown in the right-hand panel, where it can be seen that the velocity drops from ~220 m/s (at the nozzle) to about 50 m/s (~30 mm from the nozzle). To the best of the authors’ knowledge, this is the first time the velocity field of an atomizing spray has been captured using a method that is based on structured illumination.

**Figure 8:**
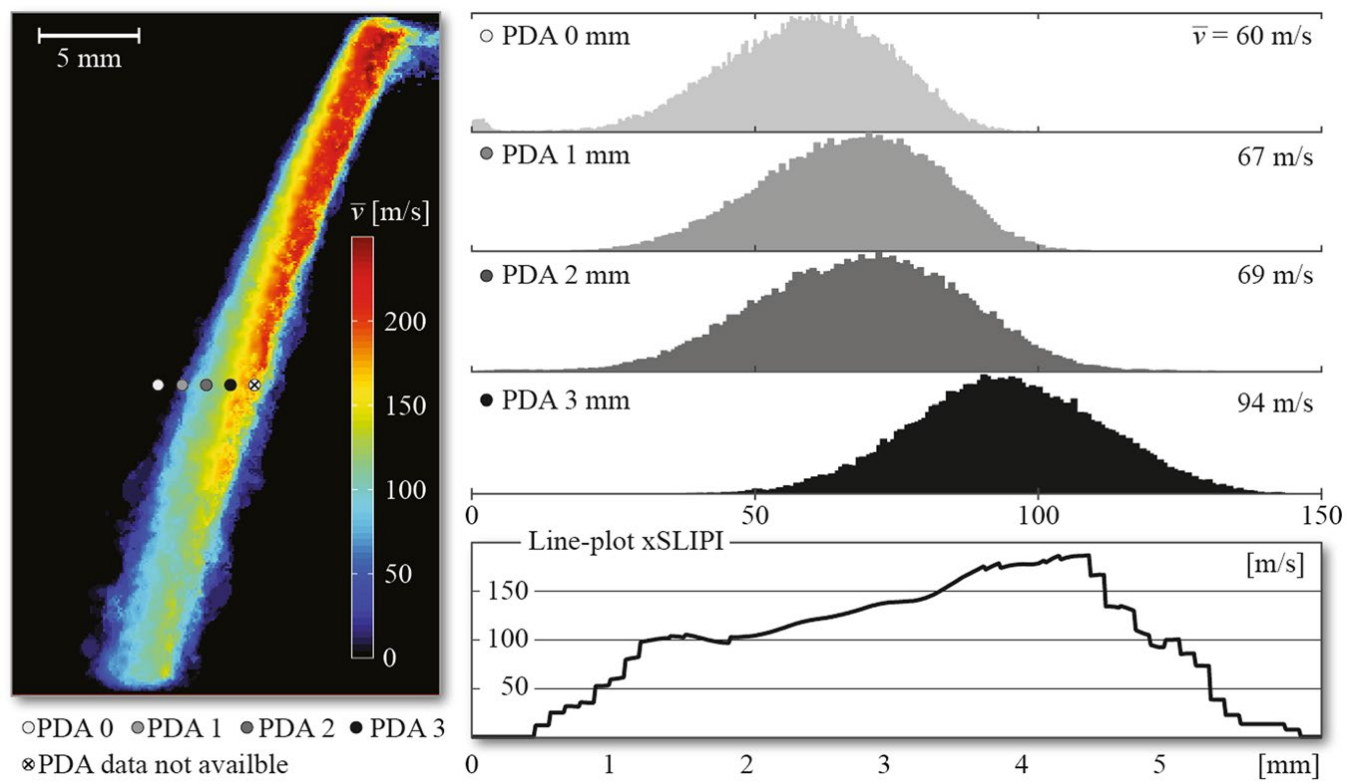
Comparison between an averaged velocity field extracted using PIV-xSLIPI and PDA data (liquid injection pressure 150 bars). (Left) Ensemble-averaged velocity field (from 15 PIV-xSLIPI measurements), where the circles mark the positions for the PDA measurements. (Right) Histograms from the PDA measurements together with an xSLIPI line-plot, extracted from the same region. Although PIV measures velocities across a large 2D field the technique is not able to extract the slower-moving droplets. This explains the divergences between the two datasets. However, similar velocity trends can be observed, with an increase of droplet velocity towards the spray center.

Figure 8 shows a comparison between ensemble-averaged PIV xSLIPI and Phase Doppler Anemometry (PDA) measurements (by using a Phase Doppler Interferometer TK2 instrument from Artium) across a line located at 18 mm from the nozzle tip. Note here that the PDA measurement was including the slower-moving droplets, whereas the accuracy of PIV, as previously mentioned, depends on the value of ∆t. Despite this technological difference, both datasets show similar velocities as well as an increase of droplet velocity towards the center of the spray.

## Conclusions

In conclusion, we have presented a novel Structured Illumination approach able to spatially resolve micrometric liquid structures moving at high velocities within an optically dense spray system. The visualization of such structures is, on its own, very challenging due to both the turbidity and the transient nature of such media. In addition, good image spatial resolution is required to be able to visualize droplets of size ranging between 10 and 100 microns. The xSI approach proposed in this article solves these measurement challenges, as it efficiently removes the contribution from multiply scattered light in a single-shot configuration with minimum loss in spatial resolution. The required optical setup to achieve these results remains fairly simple and inexpensive and can relatively easily be implemented in an already existing structured illumination setup. The key idea, here, is to illuminate the sample with two crossed intensity-modulated light fields simultaneously and to apply an image post-processing, where a non-uniform spatial frequency lock-in filter is used on each sine component in the Fourier domain. The snapshot capability of the technique opens up for the study of flow dynamics and for the analysis of fast moving objects concealed in scattering media. Finally, the xSI concept has been applied for extracting the velocity vectors of fast moving droplets. This constitutes the first implementation of structured illumination with PIV for the study of turbid, transient media.
